# Translation, cross-cultural adaptation and measurement properties of three implementation measures into Brazilian-Portuguese

**DOI:** 10.1186/s40945-023-00160-x

**Published:** 2023-03-27

**Authors:** Iuri Fioratti, Verônica S. Santos, Lívia G. Fernandes, Karina A. Rodrigues, Renato J. Soares, Bruno T. Saragiotto

**Affiliations:** 1 Masters and Doctoral Programs in Physical Therapy, Universidade Cidade de São Paulo, São Paulo, Brazil; 2 Department of Physical Therapy, Universidade de Taubaté, São Paulo, Brazil; 3 Discipline of Physiotherapy, University of Technology Sydney, Sydney, Australia

**Keywords:** Implementation Science, Acceptability, Feasibility, Measurements

## Abstract

**Background:**

To translate and cross-culturally adapt into Brazilian-Portuguese, and to test the measurement properties of the following items of implementation outcome measures: Acceptability of Intervention Measure (AIM), Intervention Appropriateness Measure (IAM) and Feasibility of Intervention Measure (FIM).

**Methods:**

This was a measurement properties study in accordance with the Consensus-based Standards for the selection of health status Measurement Instruments (COSMIN). We conducted a translation and cross-cultural adaptation of three implementation measures according to guidelines for translation and cross-cultural adaptation, then we collected information from patients who had participated in remotely delivered physical therapy treatment for musculoskeletal condition. The patients answered the translated versions of the implementation outcome measures. The measurement properties of the three implementation outcome measures were collected in a test–retest assessment, with an interval of 7 to 14 days.. The measurement properties evaluated in this study were interpretability, measured using Ceiling and Floor Effects, reliability in test–retest evaluation, measured using Cronbach’s Alpha Coefficient, internal consistency, measured using Intraclass Correlation Coefficient and construct validity, measured using Pearson Correlation.

**Results:**

We included 104 participants (76 female). The average age of the sample was 56.8 (SD 14.8) years old. The items of implementation outcome measures (AIM, IAM, and FIM) showed 66.39%, 63.11%, and 63.93% of ceiling effects. The items of implementation outcome measures showed adequate internal consistency measured using Cronbach’s Alpha Coefficient (AIM: 0.89, IAM: 0.91, FIM: 0.93) and values of Standard Error of Measurement between 5 and 10%, showing good measurement error. The results of AIM and IAM was classified as moderate reliability and the FIM as substantial reliability. In a total 96 correlations, > 75% of correlations met our prior hypothesis.

**Conclusion:**

The three Brazilian-Portuguese versions of items of implementation outcome measures had adequate internal consistency, measurement error and construct validity. The three implementation outcome measures showed moderate to substantial reliability values. The Ceiling Effect was observed in the three measures, showing maximum values ​​in more than 15% of the evaluations.


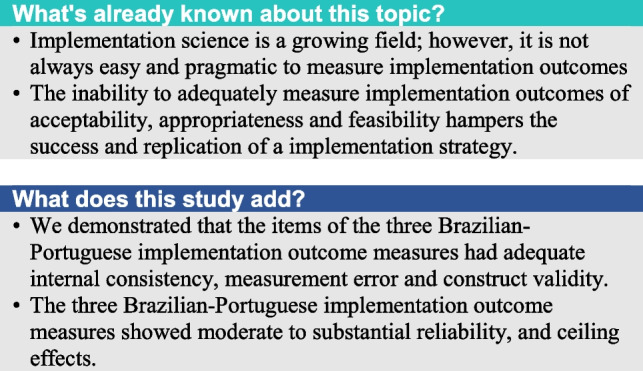


## Introduction

Implementation science is a rapidly growing field of research that aims to bring scientific findings into real-world settings and to better understand how interventions are implemented [1]. Implementation science aims to optimize the processes of development, creation, modification, testing, and application of certain health interventions, systems and procedures, following a structure based on the understanding of effectiveness, adoption, implementation, maintenance and scope [2–4]. The results of implementation research tend to be incorporated more quickly into the dynamics of health systems, due to the approximation of the reality of these systems and the anticipated understanding of the objectives necessary for a change in decision making [5].

However, in order to optimize implementation, it is crucial that we utilize validated and trustworthy measures to accurately assess the specific outcomes that will be monitored during the implementation process [6]. In implementation research, multiple outcomes are evaluated at various stages of the study, and their evaluation methods often adopt a qualitative or mixed-methods approach, instead of relying solely on quantitative measures [2, 7]. Thus, it is important to have suitable tools to gather the data that will be evaluated and to provide insights into areas of improvement, adjustment, enhancement, or optimization of a specific intervention.

One of the most important outcomes in implementation research are acceptability, appropriateness, and feasibility [4, 8]. Acceptability refers to the level of agreement and satisfaction of individual providers or consumers with the intervention. Appropriateness measures the perception of relevance or compatibility of the intervention to a specific situation or setting. Feasibility evaluates the success of implementation in a particular setting [8]. These outcomes provide insight into patients' views on proposed interventions, systems, or approaches, which can ultimately impact the effectiveness of the intervention, its adoption, and the final implementation process.

Three simple tools have recently been developed to measure acceptability, appropriateness, and feasibility, to facilitate the evaluation of these implementation outcomes [8]. The Acceptability of Intervention Measure (AIM), the Intervention Appropriateness Measure (IAM) and the Feasibility of Intervention Measure (FIM) showed adequate validity and measurement properties in a convenience sample of counselors of the American Mental Health Counselors Association about adoption of evidence-based practice [8]. However, the lack of knowledge regarding the use, validation, and translation of these tools into Brazilian Portuguese presents a significant obstacle for researchers aiming to conduct implementation research in Brazil.

The lack of translated and validated tools to measure the outcomes of acceptability, appropriateness, and feasibility makes it difficult to observe and assess these measures, hindering the ability to make necessary modifications to an intervention if results are not satisfactory for patients [3, 4]. In this way, best practices in implementation research include regular observation of results with the use of appropriate tools and intervention modifications informed by their results [4].

Thus, the aim of this study is to translate and cross-culturally adapt into Brazilian-Portuguese, and to test the measurement properties of the following implementation outcome measures: Acceptability of Intervention Measure (AIM), Intervention Appropriateness Measure (IAM) and Feasibility of Intervention Measure (FIM).

## Methods

### Study design

We conducted a measurement properties study in accordance with the taxonomy, terminology, and definitions from Consensus-based Standards for the selection of health status Measurement Instruments (COSMIN) [9]. This study was conducted after it was approved by the Human Ethics Committee of the Universidade Cidade de São Paulo (UNICID) (CAAE: 36,211,320.3.0000.0064).

### Settings and eligibility

Study patients were Brazilian adults (18 to 65 years of age) who sought physical therapy care and participated in a remotely delivered physical therapy program for the treatment of any musculoskeletal condition (e.g., low back pain, knee pain, osteoarthritis), able to read and understand Brazilian-Portuguese. Recruitment was carried out through the entry of patients into treatment and sampling for convenience in a rehabilitation center located in the state of São Paulo, Brazil. All patients participated in an individualized program in a consented manner. All patients signed an electronic consent form.

### Procedures

The study was conducted in three stages: Stage 1: Translation and cross-cultural adaptation of the AIM, IAM, and FIM. Stage 2: Data collection of patients information, and Stage 3: The measurement properties testing.

### Measurement properties of the original version

The items of the three implementation outcome measures were developed by Weiner et al. (2017) in English language, with the aim to measure the following outcomes related to implementation: acceptability, appropriateness, and feasibility [8] The three implementation outcome measures (AIM, IAM, and FIM) each consist of four items and are scored using a 5-point Likert scale, with a rating range from 1 (completely disagree) to 5 (completely agree). The final score for each instrument ranges from 4 to 20 points, with higher scores indicating higher levels of acceptability, appropriateness, or feasibility.

The measurement properties of the three proposed implementation outcome measures for translation and cross-cultural adaptation were evaluated in the original development [8]. The Cronbach alphas for the 4-item scales were 0.85 for acceptability, 0.91 for appropriateness, and 0.89 for feasibility and the results for the scales from the test–retest reliability were 0.83 for acceptability, 0.87 for appropriateness, and 0.88 for feasibility. The ICC for each construct was 0.82 (95% CI 0.63–0.94) for acceptability, 0.94 (95% CI 0.86–0.98) for appropriateness, and 0.87 (95% CI 0.72–0.96) for feasibility [8].

### Stage 1: Translation and Cross-Cultural Adaptation

For this stage, five independent steps were used:

- 1. Initial translation of the original implementation outcome measures was done by two independent translators, native in Portuguese, into Brazilian-Portuguese – one translator had no previous knowledge of implementation science.
- 2. The two translators mentioned above reached a consensus and agreed on their translations into one synthesized version.
- 3. The back-translation to the original language was done by two new translators, native in English, without prior knowledge of the objectives of the study. A summary of the back-translations was compared by same two translators, reaching a consensus of back-translation.
- 4. A committee of experts (all physical therapists with post graduated levels and over 5 years of clinical experience) produced a final version of the implementation outcome measures considering the original version, the translations, and the back translations. In this step, two specialists were introduced to the new tool and asked to check if there is consistency between the three versions. For this step, data was not requested for analysis and interpretation and possible changes of the versions was the sole responsibility of the researchers responsible for the study.
- 5. A pilot study with 30 patients was carried out to test the interpretability – ceiling and floor effects and missing data – of the implementation outcome measures.

### Stage 2: Data collection of patients information

In the second phase of the study, data was gathered from patients who participated in the recently established hybrid telehealth physical therapy program, which involved a combination of both online and in-person therapy sessions. These sessions included a face-to-face evaluation, customized home exercises, pain education, and manual therapy tailored to each patient's specific needs, such as location of pain, duration of symptoms, patient preference, and therapist expertise. After participating in the program, all patients were asked to complete the translated versions of the implementation outcome measures.

### Stage 3: Measurement Properties

In the third stage, we tested the measurement properties of the three implementation outcome measures [10].

The following measurement properties were measured:

- Interpretability: measures the degree to which scores from a questionnaire can receive a qualitative meaning. It is not a measurement property but is considered an important point to be measured [9].
- Reliability (domain): it is a domain that measures the degree to which scores from patients that have not changed are the same in different conditions [9].
  o Internal consistency: measure the degree to which items from a scale or subscale are correlated with each other [9, 10];
  p Reliability (measurement property): measure the degree to which variance in score is due to true differences between patients [9, 10];
  q Measurement error: measure the systematic and random error in scores that are not due to true changes in patients [9, 10].
- Construct validity: measures the degree to which an instrument really measures the construct that it proposes to. It is a measure based on a prior hypothesis [9, 10]. In Brazilian-Portuguese there are no similar instruments available. Therefore, we conducted the construct validity between the three implementation outcome measures correlating with each other.

A retest was requested from all participants. For the test–retest analysis, we used an interval of 7 to 14 days between the first and second responses to avoid memory bias [10].

### Statistical analysis

The online survey’s responses were exported to a Microsoft Excel sheet and all data was codified in numbers. The results were analyzed descriptively. The dichotomous variables were summarized using the frequency (n) and percentage (%) distribution. Numerical variables that have an approximately normal distribution were summarized using mean and standard deviation (SD). Numerical variables that do not have an approximately normal distribution were summarized using the median and the interquartile range of 25% to 75%. The 95% confidence interval (95% CIs) was calculated around proportions.

The measurement properties were measured as follows:

- Interpretability: it was measured by ceiling and floor effects and by missing value. Ceiling and floor effects were defined as the percentage of individuals that scored the maximum or the minimum score on the questionnaire. Ceiling or floor effects are potentially present when this percentage is 15% or more. Missing value was measured by frequency and percentage of missing values [10];
- Reliability:
  o Internal consistency: it was measured using Cronbach’s Alpha coefficient, in which values between 0.70 and 0.95 are considered appropriate [10].
  p Reliability: it was measure by Intraclass Correlation Coefficient (ICC) using a 2-way random model and absolute agreement. Reliability for ICC values of less than 0.40 was interpreted as poor, 0.40 to 0.75 as moderate, greater than 0.75 to 0.90 as substantial, and greater than 0.90 as excellent [11].
  q Measurement error: it was measure by Standard Error of Measurement (SEM), using square root of error variance from ANOVA within group analysis [10, 12]. It was used the ratio between SEM and the total score from questionnaire to interpret as follows: ≤ 5%—very good measurement error; > 5% and ≤ 10%—good measurement error; > 10% and ≤ 20%—doubtful measurement error; and > 20%—negative measurement error. Measurement error was also measured by the Smallest Detectable Change (SDC) that was calculated by formula SDC = 1.96√2SEM [10, 11].
- Construct validity: it was measured by Pearson Correlation (r) between final scores from the three questionnaires. The Pearson Correlation should be interpreted as: ≥ 0.70 – strong convergence; 0.50 to 0.69 – moderate convergence; 0.20 to 0.40 – moderate divergence; and ≤ 0.20 strong divergence [13, 14]. To confirm the construct validity 75% of the correlations need to be in accordance with prior hypothesis [10]. Our prior hypothesis was that the items from three implementation outcome measures will have moderate to strong convergence among them. All analysis was conducted using SPSS version 25.0 (IBM Corp., Armonk, NY, USA).

## Results

We included 104 participants to the cross-sectional analysis and 67 patients to the test–retest analysis from a remote physiotherapy program for the treatment of musculoskeletal conditions. A total of 76 (73%) participants were female. The average age of the sample was 56.8 (SD 14.8) years old (Table 1).

**Table 1:** Sociodemographic characteristics of the participants (*n* = 104)

Age, mean (SD)	56.8 (14.8)
Gender, *n* (%)	
Female	76 (73%)
Male	28 (27%)
Number of telehealth sessions, mean (SD)	12.3 (5.9)
Total number of sessions (telehealth and face-to-face), mean (SD)	19.6 (9.6)
Region of pain, *n* (%)	
Back	41 (33.6)
Neck	8 (6.6)
Upper limbs	11 (9.0)
Lower limbs	14 (11.5)
Multiple sites	30 (24.6)
Duration of symptoms (months), median (IQR)	12 (3:38)
Pain intensity (0–10), mean (SD)	6.0 (2.4)

*SD* Standard deviation *IQR* Interquartile range

### Translation and cross-cultural adaptation

During the process of translation and cross-cultural adaptation, only minor changes were made from the original version in English.

Minor changes were made to adapt the verb tense in sentences that were in the present to adapt to sentences in the past tense. The use of a linking verb *“ser”* was used in some sentences.

### Interpretability

The three implementation outcome measures (AIM, IAM, and FIM) showed 66.4%, 63.1%, and 63.9% of ceiling effects, respectively. It was not observed any missing data.

### Reliability

The three implementation outcome measures (AIM, IAM, and FIM) showed adequate internal consistency and good measurement error. Related to reliability the results of AIM and IAM was classified as moderate reliability and the FIM was classified as substantial reliability (Table 2).

**Table 2:** reliability of three implementation outcome measures (AIM, IAM, and FIM)

Reliability	AIM	IAM	FIM
Internal Consistency (range Alpha if item deleted)	0.89 (0.84 to 0.88)	0.91 (0.88 to 0.90)	0.93 (0.87 to 0.96)
Reliability, ICC (95% CI)	0.65 (0.44 to 0.79)	0.62 (0.38 to 0.77)	0.79 (0.65 to 0.87)
Measurement error (SEM, % related to the total score)	1.51 (7.55)	1.84 (9.2)	1.16 (5.8)
Measurement error (SDC)	4.18	5.10	3.10

*ICC, Intraclass Correlation Coefficients*, *CI Confidence Interval, SEM Standard Error of Measurement*, *SDC Smallest Detectable Change*, *AIM* *Acceptability of Interventions Measure, IAM* *Intervention Appropriateness Measure, FIM: Feasibility of Intervention Measure*

### Construct validity

The construct was adequate with 91.7% of correlation in accordance with our prior hypothesis (Table 3). We conducted 96 correlations between items from three implementation outcome measures (AIM, IAM, and FIM) and only eight correlations were less than 0.50.

**Table 3:** Correlations between the three implementation outcome measures (AIM, IAM, and FIM)

	**AIM 1**	**AIM 2**	**AIM 3**	**AIM 4**	**IAM 1**	**IAM 2**	**IAM 3**	**IAM 4**	**FIM 1**	**FIM 2**	**FIM 3**	**FIM 4**
AIM 1	**-**	**0.65**	**0.64**	**0.63**	**0.68**	**0.71**	**0.64**	**0.71**	**0.68**	**0.59**	**0.73**	0.44
AIM 2	**0.65**	**-**	**0.79**	**0.68**	**0.69**	**0.64**	**0.59**	**0.60**	**0.60**	**0.54**	**0.65**	**0.51**
AIM 3	**0.64**	**0.79**	**-**	**0.72**	**0.66**	**0.58**	**0.63**	**0.65**	**0.66**	**0.57**	**0.67**	**0.60**
AIM 4	**0.63**	**0.68**	**0.72**	**-**	**0.67**	**0.67**	**0.64**	**0.58**	**0.55**	**0.56**	**0.68**	0.46
IAM 1	**0.68**	**0.69**	**0.66**	**0.67**	**-**	**0.86**	**0.66**	**0.67**	**0.63**	**0.57**	**0.70**	0.43
IAM 2	**0.71**	**0.64**	**0.58**	**0.67**	**0.86**	**-**	**0.75**	**0.67**	**0.64**	**0.59**	**0.67**	0.41
IAM 3	**0.64**	**0.59**	**0.63**	**0.64**	**0.66**	**0.75**	**-**	**0.83**	**0.88**	**0.84**	**0.86**	**0.71**
IAM 4	**0.71**	**0.60**	**0.65**	**0.58**	**0.67**	**0.67**	**0.83**	**-**	**0.87**	**0.81**	**0.86**	**0.66**
FIM 1	**0.68**	**0.60**	**0.66**	**0.55**	**0.63**	**0.64**	**0.88**	**0.87**	**-**	**0.88**	**0.90**	**0.74**
FIM 2	**0.59**	**0.54**	**0.57**	**0.56**	**0.57**	**0.59**	**0.84**	**0.81**	**0.88**	**-**	**0.86**	**0.67**
FIM 3	**0.73**	**0.65**	**0.67**	**0.68**	**0.70**	**0.67**	**0.86**	**0.86**	**0.90**	**0.86**	**-**	**0.62**
FIM 4	0.44	**0.51**	**0.60**	0.46	0.43	0.41	**0.71**	**0.66**	**0.74**	**0.67**	**0.62**	**-**

*Correlations in agreement with the prior hypothesis in bold,* AIM Acceptability of Interventions Measure, *IAM* Intervention Appropriateness Measure, *FIM* Feasibility of Intervention Measure

## Discussion

The items of the three implementation outcome measures—AIM, IAM and FIM – were translated and cross-culturally adapted to Brazilian-Portuguese with no major changes related to the original version. The items of the three measures had adequate internal consistency, measurement error and construct validity. The AIM and IAM showed moderate reliability and the FIM showed substantial reliability to be used in Brazilian adults under remote programs for the treatment of musculoskeletal conditions.

This study had both strengths and limitations. One of its strengths is that the study was conducted in compliance with the COSMIN taxonomy, terminology, and definitions, ensuring the use of consistent language and definitions in the reporting. Another strength is that the study was carried out during the implementation of the telehealth program, allowing the participants to be in the appropriate context to answer the implementation outcome measures.

However, the study also had some limitations. One of its limitations is the limited external validity due to the lack of information on the educational or economic status of the participants, as it was not included in the baseline questionnaire of the telehealth program. Additionally, the sample was limited to one health center in one city in the state of Sao Paulo, which may not be representative of other regions or populations. The translation and cross-cultural adaptation process also posed some challenges. Due to feasibility reasons, it was not possible to conduct interviews with the participants, and the survey was administered online. Additionally, the team did not include methodologists, language professionals, or translators, limiting the quality of the translation process. However, the team consisted of health professionals who were able to provide valuable perspectives on the content of the measures.

The only previous study that have tested the measurement properties of these instruments found an internal consistency of the original version almost the same as the one found in our study, with 0.89, 0.87 and 0.89 respectively [8]. Therefore, it appears that even after being translated and adapted into Brazilian-Portuguese, the items continue to be correlated with each other. Other implementation outcome measures described in the literature assess similar outcomes in different ways, making comparison challenging. Hence, based on our research needs, the most informative data on the properties of measurements is associated with the original version of the AIM, IAM and FIM.

The findings of this study showed that three implementation outcome measures can be used in future studies and in clinical practice to measure implementation outcomes. The findings suggested that the three implementation outcome measures are able to measure stable participants with 7- to 14-day intervals and achieved the almost same results. The results related to internal consistency indicate that the items of each implementation measure evaluated are correlated with each other. In addition, evaluations show that there is little variation in the results if only one item is excluded. This means that all items contributed almost equally to the construct assessment [10]. The three implementation outcome measures showed ceiling effects, making real understanding and identification of the receipt of satisfactory results difficult.

Future studies are necessary to answers some questions. We cannot guarantee if in other health conditions and settings the measurement properties will remain stable. Therefore, we suggest conducting the measurement properties in other health conditions and in healthy people, and in other settings. It is also necessary that further specific analysis is conducted, such as Confirmatory Factor Analysis.

## Conclusion

The items of the three Brazilian-Portuguese versions of implementation outcome measures (AIM, IAM, and FIM) had adequate internal consistency, measurement error and construct validity. The three implementation outcome measures showed moderate to substantial reliability, and ceiling effects.

## Data Availability

All data shown in this study are real and are available to editors for questions and conferences.

## Abbreviations

AIM: Acceptability of Interventions Measures
IAM: Intervention Appropriateness Measure
FIM: Feasibility of Intervention Measure
COSMIN: Consensus-based Standards for the selection of health status Measurement Instruments
SEM: Standard Error of Measurement (SEM)
SDC: Smallest Detectable Change (SDC)
